# Recent Progress on Plant-Inspired Soft Robotics with Hydrogel Building Blocks: Fabrication, Actuation and Application

**DOI:** 10.3390/mi12060608

**Published:** 2021-05-24

**Authors:** Zhenyu Xu, Yongsen Zhou, Baoping Zhang, Chao Zhang, Jianfeng Wang, Zuankai Wang

**Affiliations:** 1 Department of Mechanical Engineering, City University of Hong Kong, Kowloon, Hong Kong 999077, China; zhenyuxu4-c@my.cityu.edu.hk (Z.X.); yongszhou2-c@my.cityu.edu.hk (Y.Z.); baozhang@cityu.edu.hk (B.Z.); czhan25@cityu.edu.hk (C.Z.); 2 Department of Architecture and Civil Engineering, City University of Hong Kong, Kowloon, Hong Kong 999077, China; 3 Center for Nature-Inspired Engineering, City University of Hong Kong, Kowloon, Hong Kong 999077, China

**Keywords:** plant-inspired, soft robotics, adaptive, stimuli-responsive, hydrogel

## Abstract

Millions of years’ evolution has imparted life on earth with excellent environment adaptability. Of particular interest to scientists are some plants capable of macroscopically and reversibly altering their morphological and mechanical properties in response to external stimuli from the surrounding environment. These intriguing natural phenomena and underlying actuation mechanisms have provided important design guidance and principles for man-made soft robotic systems. Constructing bio-inspired soft robotic systems with effective actuation requires the efficient supply of mechanical energy generated from external inputs, such as temperature, light, and electricity. By combining bio-inspired designs with stimuli-responsive materials, various intelligent soft robotic systems that demonstrate promising and exciting results have been developed. As one of the building materials for soft robotics, hydrogels are gaining increasing attention owing to their advantageous properties, such as ultra-tunable modulus, high compliance, varying stimuli-responsiveness, good biocompatibility, and high transparency. In this review article, we summarize the recent progress on plant-inspired soft robotics assembled by stimuli-responsive hydrogels with a particular focus on their actuation mechanisms, fabrication, and application. Meanwhile, some critical challenges and problems associated with current hydrogel-based soft robotics are briefly introduced, and possible solutions are proposed. We expect that this review would provide elementary tutorial guidelines to audiences who are interested in the study on nature-inspired soft robotics, especially hydrogel-based intelligent soft robotic systems.

## 1. Introduction

As Charles Darwin once said, it is not the strongest of the species that survives, nor the most intelligent; it is the one most adaptable to change [1]. Through millions of years’ evolution, lives on the earth have developed basic and crucial surviving skills: environment adaptability. Having sensed different environmental signals (e.g., seasonal, daily, and instant change of temperature, illumination, and humidity), many animals can actively respond with actions of biological importance. For instance, birds, such as storks, turtle doves, and swallows, migrate between breeding and non-breeding grounds [2]. Atlantic salmon, a well-known anadromous fish, returns to its home streams and rivers to spawn after a long migration in the ocean, ensuring the survival of its offspring [3].

Plants can demonstrate movements from a slow to fast, and even to furious speed, even though their roots are fixed to the ground [4,5,6]. Different from the muscular movements in animals, movements in plants are often stimuli-responsive and can be generally categorized into two types: tropic movements and nastic movements [7,8]. Tropic movements, associated with the growth of plants, are universal in plants. For instance, the phenomenon of roots growing toward the gravitational pull, while shoots growing against the gravitational pull is called gravitropism. Similarly, there are also phototropism and hydrotropism, which are both beneficial to the growth of plants. Unlike the universal and directional tropic movements, nastic movements of plants are more individualistic and independent of the direction of the stimulus [8]. In response to changes in relative humidity, pinecone [9] opens/closes its scales to release its seeds, while wheat awn [10] does so by bending/unbending its seed dispersal units. Instead of responding to the humidity change, ice plant opens its protective valves to release the seeds only when the seed capsule is sufficiently hydrated with liquid water, which ensures that the seeds are dispersed under favorable conditions for germination [11]. Another fascinating nastic movement of plants is the folding/unfolding response to external mechanical stimulus (e.g., touch or vibration). For instance, Venus flytrap can rapidly close its trap within 100 ms when its sensitive trigger hairs are stimulated by the struggle of insects and reopens its trap when the food is fully digested [12]. Similar to Venus flytrap’s pray capturing technique, sundew uses its sticky glands and wrappable tentacles to trap the visiting insect and unwrap its tentacles for the next round of hunting [13]. Another well-known plant responsive to the mechanical stimulus is mimosa [14]; it folds its leaves inward to defend itself from potential harm when being touched or shaken and unfolds its leaves a few minutes later when the danger is vanished. In addition to the hydronastic (i.e., humidity-/water-responsive) movements and thigmonastic (i.e., mechano-responsive) movements in plants, there are some other types of nastic movements, like photonastic and thermonastic movements, in flowers [15]. Notably, these nastic movements in plants are energy-efficient, fast, reversible, and robust, representing perfect modeling systems for the design of artificial soft robotics [16,17].

By combining bio-inspired designs with stimuli-responsive motifs, numerous intelligent soft robotic prototypes have been developed [18]. These intelligent biomimetic soft systems are finding applications in shape morphing, actuation, and gripping/manipulation where mechanical forces are effectively generated from various energy inputs, such as heat, electricity, and illumination. Soft robotic systems are normally fabricated by compliant soft materials, such as silicone elastomers, polyurethanes, hydrogels, braided fabrics, hydraulic fluids, and gasses [18]. Among these building materials, hydrogels composed of up to 90% of water embedded in polymeric networks are standing out because of the excellent integration of important properties, including being variable moduli-matching biological soft systems, stimuli-responsiveness available, stretchable and tough, non-toxic, conductive, and transparent [19]. More importantly, fabrication of hydrogel-based soft robotics is greatly facilitated by the facile UV polymerization and 3D printing of hydrogels [20,21,22].

**Figure 1:**
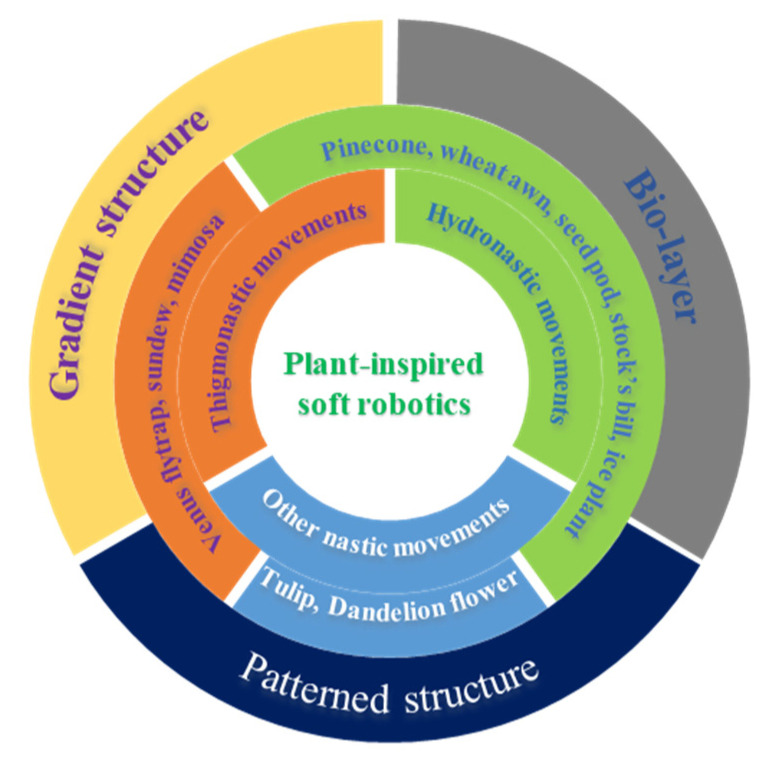
Plant-inspired soft robotics: nastic movements in plants and derived design principles for biomimetic soft robotics.

In this review article, we aim to summarize recent progress on the development of hydrogel-based soft robotics with plant-inspired designs and actuation mechanisms. The article is organized in the following way (Figure 1). First, the two common types of nastic movements of plants, namely hydronastic movements and thigmonastic movements, are introduced in Section 2. Then, the underlying actuation mechanism for each type of nastic movement and the derivation of the design principles for biomimetic soft robotics are discussed. Based on these bio-inspired design principles, representative prototypes with stimuli-responsive hydrogels as the building materials in terms of fabrication and actuation mechanism are introduced in Section 3. Finally, some critical challenges hampering the development of hydrogel-based soft robotics are discussed, and the corresponding possible solutions are proposed in Section 4. It is anticipated that this review article would spark broader interests in the investigation and advancement of biomimicry hydrogel-based soft robotics.

## 2. Nastic Movements of Plants

Nastic movements of plants have unique characteristics, such as stimuli-responsiveness independent of the direction of stimuli, reversibility, and quick response attributed to the specific actuation mechanisms. In this section, we introduce two types of common nastic movements in plants: hydronastic movement responding to humidity and thigmonastic movement responding to touch.

### 2.1. Hydronastic Movements and Actuation Mechanisms

In the wild nature, weather could change unexpectedly at any time. Daily humidity change is one of the major changes that plants experience in the course of their growth. In response to this humidity change, plants with seed dispersal units release their seeds to the ground for the purpose of reproduction [23,24]. During the seed releasing process, the dispersal units experience slow-to-fast and humidity-responsive actuations known as hydronastic behaviors, such as bending, twisting, coiling, and folding, providing excellent models for the design and fabrication of soft robotics [25]. In the following subsections, we introduce the humidity-triggered actuation mechanisms in representative plants bearing seed dispersal units, including pinecone, wheat awn, seed pod, stork’s bill, and ice plant.

#### 2.1.1. Pinecone

**Figure 2:**
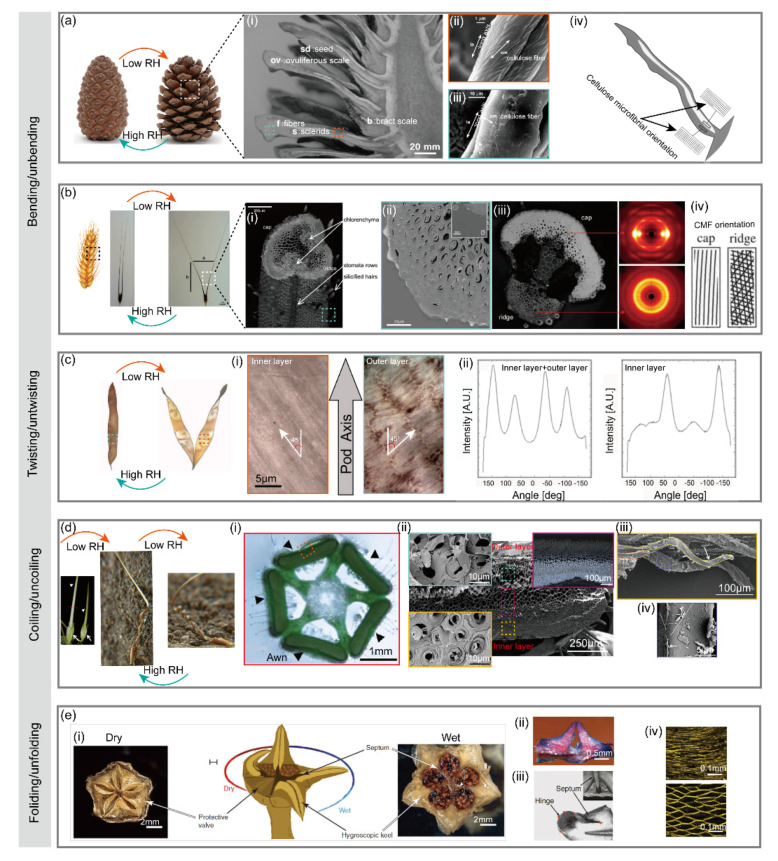
Hydronastic movements in plants and actuation mechanisms. (**a**) Bending/unbending of pinecone scale (reproduced with permission from Reference [9]). (**b**) Bending/unbending of wheat awn (reproduced with permission from Reference [10]). (**c**) Twisting/untwisting of seed pod (reproduced with permission from Reference [27]). (**d**) Coiling/uncoiling of stork’s bill (reproduced with permission from Reference [31]). (**e**) Folding/unfolding of ice plant valve (reproduced with \permission from Reference [11]).

The seed-bearing scales of pinecone plants disperse the seeds by opening the scales at dry conditions and keep the scales closed at wet conditions [26]. Notably, the opening and closing of scales can be repeated many times at alternating humidity levels. Such reversible opening/closing behavior is highly related to the structural assembly of the scales rather than cellular activity [9]. Pinecone has two types of scales: larger ovuliferous scale and smaller bract scale Figure 2a(i). The ovuliferous scale responds to humidity change, while the bract scale is humidity-inert. Located in the inner surface of the ovuliferous scale are bundled sclerenchyma fibers (8–12 mm in diameter and 150–200 mm long), while the outer surface are sclerids (20–30 mm diameter and 80–120 mm long), as shown in Figure 2a(i). The ovuliferous scale moves towards the center of the cone in high relative humidity and away from the center in low relative humidity, generating a bending angle up to 55°. Such a drastic change of bending angle during the opening/closing process of pinecone scales is believed to be associated with the orientation difference of cellulose microfibrils (CMFs) in the inner fibers and outer sclerids [9,27]. As shown in Figure 2a(ii), Figure 2a(iii), the angle of winding of microfibrils (θ) relative to the long axis of the cell in sclerids (74 ± 5°) is much higher than that of fibers (30 ± 2°). This winding angle difference results in different coefficients of hygroscopic expansion of the cells in fibers (0.06 ± 0.02) and sclerids (0.20 ± 0.04). The higher hygroscopic expansion coefficient of outer sclerids enables the longitudinal expanding/shrinking of ovuliferous scales at wet/dry conditions, while the inner fibers with lower hygroscopic expansion coefficients do not respond as strongly as the outer sclerids to humidity change but resist the expanding/shrinking [28,29,30]. Therefore, the key to opening/closing of pinecone scales lies in the CMFs with different orientations Figure 2a(iv), leading to anisotropic expanding/shrinking while being resisted [9,27,28].

#### 2.1.2. Wheat Awn

Wild wheat has bundles of seed dispersal units composed of two pronounced awns that balance the unit as it falls to the ground. The wheat awn plays a crucial role in the seed dispersing process. The awns bend inward when the air is wet (at nighttime) and bend outward to release seeds when the air is dry (at daytime). The bending process is reversible as humidity changes alternatively. The underlying humidity-responsive and reversible actuation mechanism is related to the special structural configuration of the awn [10]. The awn has a wide cap and a relatively narrow ridge facing to the same direction along the awn Figure 2b(i). Though the cap and the ridge show no significant difference in chemical composition, they display distinct stiffnesses as revealed by scanning acoustic microscopy Figure 2b(iii) and nanoindentation test. The cap has a much higher local effective Young’s modulus (20.5 ± 2.6 GPa) than that of ridge (10.0 ± 2.8 GPa). Such stiffness variation is mainly attributed to the orientation of cellulose microfibrils (CMFs) or microfibril angle (MFA). As revealed by the wide-angle X-ray scattering, the cap has an MFA close to 0, meaning that the CMFs are well-aligned along the axis of awn. However, the CMFs in the ridge are randomly oriented Figure 2b(iv). Notably, such difference of CMF orientation only occurs in the lower part of the awn (2 cm below the seed). Though the humidity-responsive actuation of wheat awn resembles that of pinecone scale [9], there is still a slight difference in the specific actuation mechanism. In response to the humidity rise, the cap region expands laterally, while the ridge region expands longitudinally, pushing the awns together. When the humidity drops, the region contracts, pulling the awns apart.

#### 2.1.3. Seed Pod

Bauhinia seed pod remains closed at wet conditions yet opens to release the seeds at dry conditions [27,32]. The seed pod has two pod valves which are initially flat at wet conditions. When the air is dry, the pod valves twist into helical strips of opposite handedness, during which the pod opens to release the seeds. The seed dispersal process is, therefore, a chirality-creating process [32,33]. The underlying mechanism behind this flat-to-helical transition during pod opening is again associated with the orientation of cellulose microfibrils (CMFs) in pod valves [27,32]. Oriented at an angle of +45° and −45° with respect to the longitudinal axis of the pod Figure 2c(i), CMFs in the inner and outer layers of the pod valve act as a scaffold resisting the contraction of the round parenchyma cells in which they are embedded. When the pod gets dried by the air with a low humidity, the two juxtaposing fibrous layers shrink perpendicularly to its orientation, inducing mirror-image saddle-like curvatures an eventually a dramatic opening [34]. Note that such twisting behavior of pod valves can turn into coiling when the ratio between the valve’s width and thickness exceeds a certain value, which is seen in the pod of Bauhinia galpinii [35].

#### 2.1.4. Stork’s Bill

Erodium gruinum (also known as stork’s bill) is one of the species in the Geraniaceae plant family featured with beak-like fruits that are composed of five seeds appended by long tapering awns [36]. Prior to ripening, five awns are connected to a central column in one fruit (Figure 2d(i)). As the seeds mature, the awns dry out and contract. In this process, elastic energy builds up to the breaking point. The extreme torque generated by the twisting awn catapults the ripened seeds away from the parent plant [37]. Once on the ground, the awns respond to humidity and quickly change its shape with the assistance from the hygroscopic cells. The awn would coil tightly at low humidity and uncoil at high humidity. Such an alternating coiling/uncoiling process helps the awn to locomote on the ground until a safe germination site is found [38]. The underlying mechanism for this reversible coiling/uncoiling is different from the bending wheat awn and twisting pod valve where a bilayer structure is involved. Instead, a specialized single layer consisting of a mechanically uniform tissue is responsible for the hygroscopic coiling/uncoiling movement of stork’s bill. Though the awn also has an inner layer and an outer layer with different morphologies Figure 2d(ii), each layer displays similar coiling behaviors even when they are split lengthwise along the awn [31]. Revealed by scanning electron microscopy, the inner layer of the awn shows a group of coiling cells behind a single coiled cell connected to the tissue at one end Figure 2d(iii). Furthermore, cryo-scanning electron images show the change in microfibril angle in a single cell Figure 2d(iv). Therefore, coiling of the stork’s bill awn arises from an intrinsic property of the cells in the inner layer. Single cells cooperate with each other to produce a macroscopic coil. As the awn dries, its main longitudinal axis is bending and twisting at the same time. This distortion is manifested as coiling of the entire bundle of cells [31,36,39].

#### 2.1.5. Ice Plant

Living in arid and semi-arid habitats, the Aizoaceae (also known as ice plant) has developed a unique seed dispersal strategy different from other plants [40]. Rather than releasing the seeds at dry conditions, ice plant has protective valves surrounding the seed capsules. The valves remain folded when the air is dry and will only unfold to release the seeds when they get hydrated by water (from rain) [41]. Such a folding/unfolding behavior in ice plants is supported by a different mechanism from the bending, twisting, and coiling mechanism mentioned previously. Responsible for the folding/unfolding valves of ice plant is the sophisticated hierarchical structure of the seed capsules, which allows for a more complex origami-like unfolding movement that incorporates an effective flexing-and-packing mechanism [11]. The seed capsule of ice plant is partitioned by five septa lying beneath the center of each valve Figure 2e(i). Buried along the center of inner valve surface is a hygroscopic keel consisting of two separated halves Figure 2e(i). As shown in Figure 2e(ii), the two halves only come into contact when hydrated, creating a bridge-like structure with an empty space between the upper portion of the keel and valve backing [11]. At dry conditions, the five valves curve upwards to lock the hygroscopically active hinge Figure 2e(iii). With rain, the valves straighten, the hinge hydrates and the compact tissue in its inner side expands. The cells pre-arranged as a collapsed honeycomb swell to form an open honeycomb structure Figure 2e(iv). Such expansion is attributed to the highly hygroscopic cellulosic inner layer in the cell wall. The extension of the inner layer in relation to the outer backing resistance layer pushes the valve open. Thus, the capsule opens to disperse the seeds by the striking raindrops. Once dry again, the hinges bend to curl the valves, locking the capsule until the next rain comes. The specialized tissue involved in the construction of the hinges allows this movement to repeat with little distortion [42].

### 2.2. Thigmonastic Movements and Actuation Mechanisms

In addition to the hydronastic movement involved in the seed dispersal process of plants, there is another kind of nastic movement of plants that responds to external mechanical touch: thigmonastic movement. Plants evolve with the build-in thigmonastic movements primarily for the purpose of prey-capturing and self-defending. In this section, we introduce three representative plants with fascinating thigmonastic movements: Venus flytrap, sundew, and mimosa.

#### 2.2.1. Venus Flytrap

**Figure 3:**
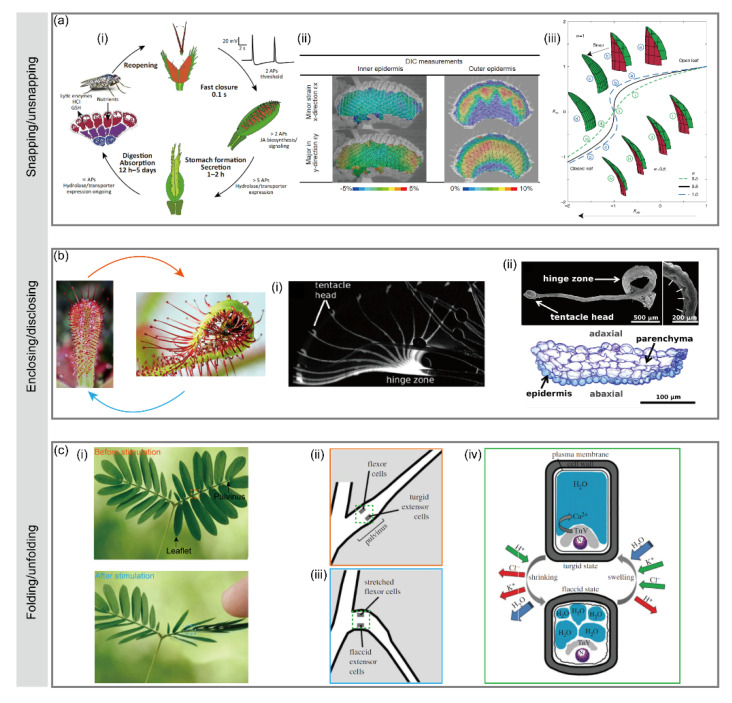
Thigmonastic movements in plants and actuation mechanisms. (**a**) Snapping/unsnapping of Venus flytrap (reproduced with permission from References [12,43,47]). (**b**) Enclosing/disclosing of sundew tentacles (reproduced with permission from Reference [52]). (**c**) Folding/unfolding of mimosa leaflets (reproduced with permission from Reference [53]).

Living in poor soil, the carnivorous plant Dionaea muscipula (also known as Venus flytrap) gets nutrients from captured preys by its touch-sensitive bivalved leaves [43]. Protruding from the upper leaf epidermis are six tiny hairs known as trigger hairs Figure 3a(i). Once these highly sensitive trigger hairs have been continuously stricken by any visiting insect, an electrical impulse is triggered, causing the leave to snap shut within 0.1 s [12]. After the prey is digested by the secreted enzyme, the trap reopens for the next hunting. This rapid snaping behavior induced by mechanical stimulation is driven by the propagation of action potentials in the leaves [44,45,46,47], which results in a simultaneous expansion of the outer epidermis and a lower shrinkage of the inner epidermis Figure 3a(ii). Though the underlying bio-mechanical principles are not yet fully understood [48], the snap-bulking instability Figure 3a(iii) is involved in the trapping mechanics where an elaborate interplay between swelling/shrinking processes of the various tissue layers and the release of trap-internal prestress is involved [12,48,49,50,51]. Specifically, the traps are in a state of ready-to-snap which is enabled by the internal hydraulic pressure differences between layers (i.e., prestress) before it is mechanically stimulated. Once the snapping is triggered, the prestress is released.

#### 2.2.2. Sundew

Another carnivorous plant living in nutrient-poor soil is sundew. It also has a prey-capturing organ featured with long tentacles protruding from its leaf, each having a sticky gland at the tip [49]. These glands produce nectar to attract small insects. Once stuck by the viscous adhesive, the insect is trapped by the closing leaf with fibrillar tentacles. During this enclosing process, chemical substances, such as jasmonates, are accumulated, triggering the bending of sundew leaves [13]. It is later revealed that recepwtor potentials and a series of action potentials are generated in the tentacles by the mechanical stimulation [50], which causes the bending to trap the insect Figure 3b(i). Importantly, chemical substances from the entrapped insect trigger the accumulation of jasmonates and digestive enzymes [50,51]. Unfortunately, the mechanical principles underlying the trapping of sundew tentacles remain poorly understood. Two possible mechanisms have been proposed [52]: (1) rapid water transport from cells in the adaxial half of the tentacle to cells in the abaxial half, resulting in adaxial contraction and abaxial extension Figure 3b(ii); (2) a sudden loss of turgor pressure in adaxial cells followed by tentacle bending owing to prestress of the abaxial surface to release the elastic energy stored in the epidermal cells on the adaxial side.

#### 2.2.3. Mimosa

Mimosa pudica plant has very sensitive leaves. A gentle touch on the leaf would trigger the self-defending response in the plant: rapid folding up its leaves (or leaflets as they are small). Located at the base of each leaflet is a pulvinus (Figure 3c(i)) responsible for the rapid and touch-sensitive folding behavior [54]. Pulvinus contains cells full of water which pushes against the cell walls, during which turgor pressure is generated. More specifically, the pulvinus has two groups of specialized parenchymatous cells called motor cells arranged in two opposite zones Figure 3c(ii/iii): the flexor or ventral zone and the extensor or dorsal zone [55]. These motor cells can rapidly change their volume and shape induced by changes in turgor pressure. As shown in Figure 3c(iv), upon mechanical stimulation of the plant, an action potential is initiated which propagates a signal from the site of the stimulus to the pulvinus. This results in another action potential which initiates the differential transport of K^+^ and Cl^−^, leading to a change in turgor pressure in the cells. Consequently, the water potential in the extensor cells increases, resulting in loss of water and shrinkage of the cells. Whereas the water potential in the flexor cells drops, leading to swell of cells to a turgid state. As a result, the leaflet folds up [53,55].

## 3. Plant-Inspired, Hydrogel-Based Soft Robotics

**Scheme 1:**
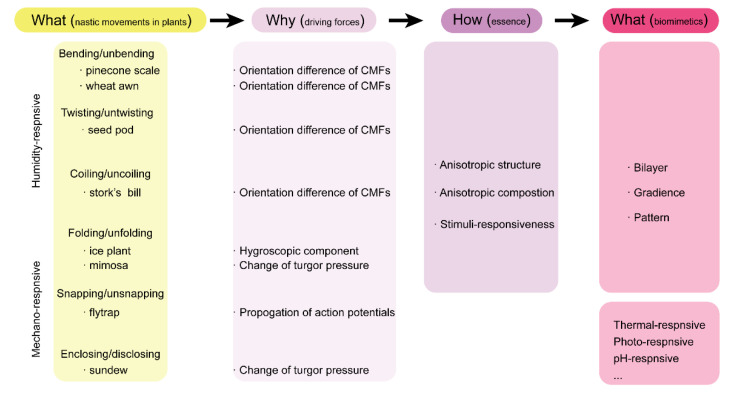
Summary of the movements of nastic movements of plants and corresponding hydrogel-based biomimetics.

As discussed above, the various movements of plants are directly triggered by change of humidity and vibrational touch. Owing to the structural and compositional differences in the moveable units of plants, response divergence in terms of motion degree and sequence (timescale) is generated, resulting in macroscopic movements. Therefore, creating anisotropy structurally or compositionally can be one of the design fundamentals of artificial soft robotics. As shown in Scheme 1, three representative design principles with build-in anisotropy can be derived to fabricate artificial soft robotics: bilayer structures, gradient structures and patterned structures [56]. Incorporating these plant-inspired design principles into stimuli-responsive hydrogels has enabled the great development of hydrogel-based soft robotics. Importantly, these hydrogel-based soft robotics can respond to more external stimuli, such as thermal, photo, and pH. In this section, we discuss the recent progress on plant-inspired soft robotics composed of hydrogel building materials.

### 3.1. Bilayer Hydrogel-Based Soft Robotics

Traditionally, bilayer hydrogel-based soft robotics, consisting of two hydrogel sheets with different swelling rates or ratios, have been successfully developed to perform controllable deformations, such as bending and bucking, on the basis of asymmetrical responsive properties of the two parts. Several approaches, including layer-by-layer polymerization, assembly of different hydrogels via reversible switches, such as host guest interactions and hydrogen bonding, have been explored to construct bilayer hydrogel actuators.

**Figure 4:**
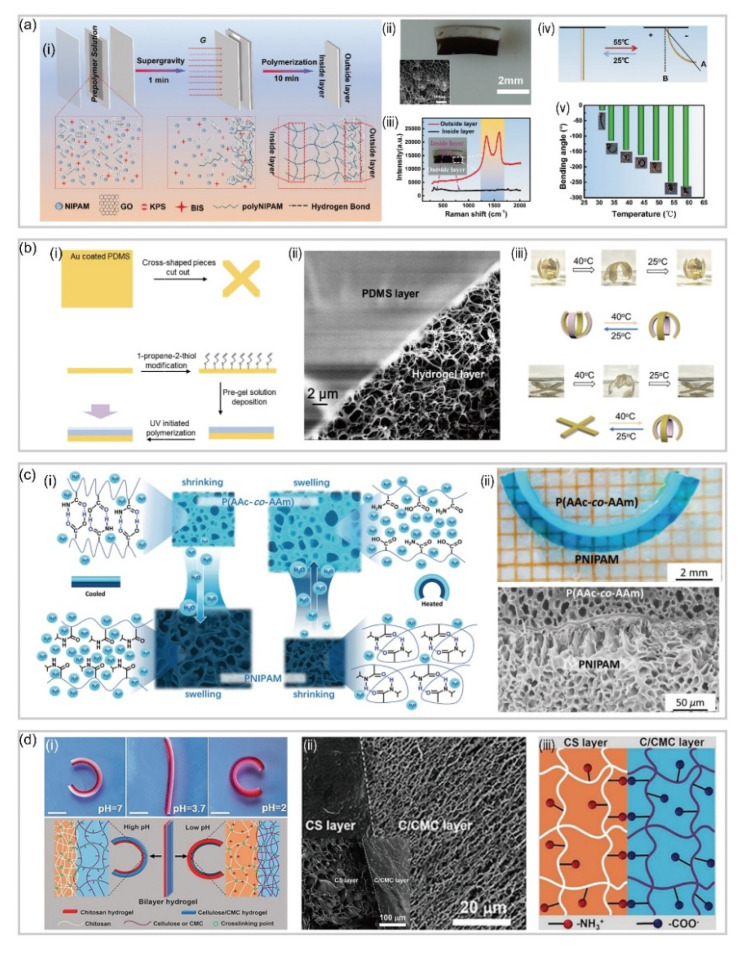
Bilayer-based hydrogel soft robotics. (**a**) Bilayer poly (N-isopropylacrylamide)/graphene oxide (pNIPAm/GO) hydrogel actuator (reproduced with permission from Reference [58]). (**b**) Temperature and pH responsive hydrogel actuator and its SEM image of the interface of the two layers (reproduced with permission from Reference [62]). (**c**) The microstructure and deformation mechanism of mimosa-inspired hydrogel actuator and SEM image showing the bilayer structure tightly joined by a 5μm interfacial layer (reproduced with permission from Reference [63]). (**d**) Schematic self-bending deformation of the pH responsive bilayer hydrogel actuator and the SEM image of the cross-section of the bilayer hydrogel (reproduced with permission from Reference [64]).

Normally, engineers tend to utilize layer-by-layer polymerization for fabricating hydrogel-based soft robotics with bilayer structures [57]. To be more specific, in the preparation process, the monomer solution of the second layer slightly penetrates into the first layer, leading to the formation of an interpenetrating-network at the interface, which acts as a junction layer to connect the two layers tightly. For instance, He et al. [58] reported a bilayer poly (N-isopropylacrylamide)/graphene oxide (pNIPAm/GO) hydrogel capable of achieving tunable, fast and bidirectional bending under a thermal or near-infrared radiation (NIR) stimulus (Figure 4a). By tuning the GO concentration and centrifugation speed, a transparent poly NIPAM layer and a dark-brown GO-rich layer are formed Figure 4a(ii), where each layer displays distinct network structures and swelling behaviors.

Different from traditional hydrogel networks which are held together by covalent crosslinks, another series of hydrogels called interpenetrating polymer networks (IPNs) are formed by combining two polymers (with at least one being responsive) that physically interact with each other to hold the network together [59]. Furthermore, there are semi-IPNs that are composed of a covalently crosslinked hydrogel infused with another linear polymer which is physically interpenetrated inside the network [60]. Compared with single network hydrogels, hydrogels with IPNs are possible to produce “new materials” under the work of each component exhibiting new behavior that is not expected from the responses of the individual components [61]. For example, by generating a poly(N-isopropylacrylamide)-based hydrogel in the presence of positively charged polyelectrolyte poly(diallyl dimethylammonium chloride) (pDADMAC) on a layer of gold-coated polydimethylsiloxane (PDMS), Li et al. [62] fabricated a temperature and pH responsive semi-IPN hydrogel-based bilayer actuator (Figure 4b). Notably, the image in Figure 4b clearly shows the crosslinked hydrogel network attached to the PDMS surface, and the two layers do not separate, even after many rounds of bending and unbending.

In addition, the layer-by-layer polymerization can also be used to fabricate bilayer hydrogels with different functions. For example, Zhen et al. [63] reported a mimosa-inspired bilayer hydrogel actuator which can function in multi-environment conditions (Figure 4c). Featured with a reverse thermal responsive bilayer composite structure, this hydrogel-based actuator is composed of a layer derived from a polymer with a lower critical solution temperature (LCST) and a second layer with an upper critical solution temperature (UCST). After heating, this bilayer hydrogel actuator could transfer water molecules from the LCST layer to the UCST layer. After cooling, the opposite process takes place, allowing for the actuation even in non-aqueous environments. Naturally, hydrogel actuators meet the extreme demand for biomedical applications due to their excellent biocompatibility and biodegradation. Duan rt al. [64] have presented a bilayer-based hydrogel actuator inspired by the bilayer structure of plant organs (Figure 4d), which is fabricated by adding cellulose to the CS and cellulose/carboxymethylcellulose (C/CMC) pre-gel alkaline aqueous solutions. By strong electrostatic attraction and chemical crosslinking, this hydrogel actuator shows a remarkable adhesion between two layers, which is about 20 kPa in tensile test.

Though bilayer hydrogel-based soft robotics have been widely investigated, there are still some challenges. The main challenge is that they could only undergo simple shape deformations, such as bending, since the hydrogels are typically isotropic materials which usually exhibit uniform volumetric expansion and contraction in response to stimuli.

### 3.2. Gradient Hydrogel-Based Soft Robotics

**Figure 5:**
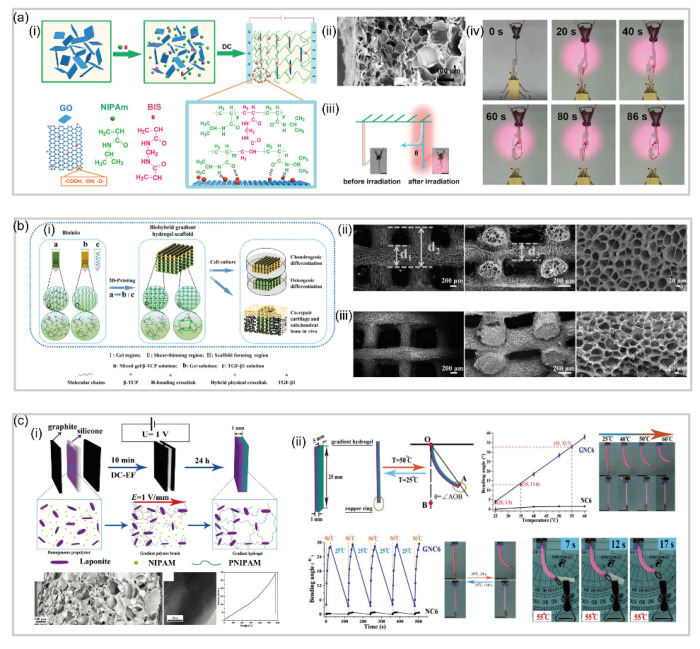
Gradient-based hydrogel soft robotics. (**a**) Photo/thermal-responsive PNIPAm/GO gradient hydrogel actuator (reproduced with permission from Reference [67]). (**b**) Thermal-assisted extrusion 3D printing of the biohybrid gradient scaffolds for repair of osteochondral defect and SEM images of the printed porous hydrogel scaffolds (reproduced with permission from Reference [68]). (**c**) Thermal-responsive pNIPAm/Laponite hydrogel actuator (reproduced with permission from Reference [71]).

Gradient hydrogel is another inhomogeneous structure to expand the application of hydrogels since it is efficient to produce complex shape deformations. One of the most common practices is to embed stimuli-responsive nanoparticles into the gradient hydrogel and utilize the migration of nanoparticles under external electric or magnetic fields during the polymerization process [56]. For example, Yang et al. [65,66] reported a series of gradient hydrogels inspired by the bilayer structures of plant organs (Figure 5a). They used the facile electrophoresis method and NIR-induced fabrication method to successfully create gradient-based hydrogel actuators. Upon near-infrared light irradiation, the hydrogel exhibits comprehensive actuation performance as a result of directional bending deformation and high photothermal conversion efficiency of graphene oxide embedded in the poly (N-isopropylacrylamide) hydrogel. Furthermore, gradient hydrogel-based soft robotics can also be fabricated by an asymmetric distribution of polymer chains. For instance, Maeda et al. [67] reported a self-walking hydrogel-based actuator without nanoparticles. In their study, two different surfaces of plates, namely a hydrophilic glass surface and a hydrophobic surface (i.e., Teflon), were used to induce uneven distribution of materials based on the difference in hydrophilicity and hydrophobicity during the polymerization.

It is worth noting that the emerging 3D printing technique is another efficient method to fabricate gradient hydrogel-based soft robotics. For instance, Fei et al. [68] presented a new bio-ink fabricated by one-step copolymerization of dual hydrogen bonding monomers, N-acryloyl glycinamide and N-[tris(hydroxymethyl)methyl] acrylamide. The as-printed hydrogel has excellent mechanical properties of high tensile strength (up to 0.41 MPa), large stretchability (up to 860%) and high compressive strength (up to 8.4 MPa). It is obvious that 3D printed gradient hydrogels are more likely to be applied to the repair of biological tissues due to its capability for accommodating tailoring structures [69,70]. Besides, engineers prefer to fabricate hydrogels with shorter response time and higher mechanical properties to be suited to more specific scenarios. For instance, Yun et al. [71] fabricated a temperature-response poly (N-isopropylacrylamide)/Laponite (pNIPAm/Laponite) gradient nanocomposite hydrogel actuator by using a facile electrophoresis method. The actuator exhibits a rapid (20 s response time) and reversible response, as well as large deformation (bending angle of 231°), which is due to the graded forces generated by the thermo-induced anisotropic shrinkage and extension of the gradient hydrogels.

### 3.3. Patterned Hydrogel-Based Soft Robotics

Patterned hydrogel is a result of the exploration of hydrogels with anisotropic structures in plane to create complex 3D structures, which achieves anisotropy by adding different patterns on the hydrogel sheet. For instance, Jasmin et al. [72] reported an enzyme-triggered hydrogel which mimics the structure of Venus fly trap. In their study, a class of self-folding hydrogel actuators which can respond to specific stimuli, such as matrix and cells at a low concentration, was fabricated. Specifically, the actuator has a bilayer configuration with a bottom passive layer and a top active layer composed of two alternating layers. When the flat hydrogel actuator is put in water, the bottom layer tends to swell more than the top layer as it is more hydrophilic. At this point, the top layer is still stiff, restricting the premature deformation. Only when the collagenase enzyme is added to the water can the stiff top layer be softened, which facilitates the swollen C layer to fold over the top layer and results in the shape transformation from a sheet to a specific shape Figure 5a(ii).

Furthermore, Wu et al. [73] reported a patterned hydrogel-based sheet in which 3D shape transformation is achieved. This hydrogel-based sheet has periodic stripes with different compositions that are arranged at an oblique angle with respect to the long axis of the sheet. These different stripes have large differences in swelling/shrinking ratios and mechanical moduli, which is responsible for the transformation from a plant sheet to a helix when it is exposed to temperature stimuli.

Though fabricating hydrogels with versatile patterns by 3D printing has become an emerging method [74,75], the typical stepwise layer-by-layer process has severely limited the printing speed. Featured with a quick 2D to 3D transformation induced by the controllable uneven stress distribution, 4D printing has become a better alternative to fabricate pattern-based hydrogel soft robotics. For instance, Huang et al. [76] fabricated a series of thermal-responsive hydrogel sheets with various patterns, which achieves complicated configuration change. They fabricated a flat hydrogel sheet which has concentric circular print layout with different exposure time of 12 and 24 s, respectively. Upon swelling by heating above 45 ℃, the printed flat sheet transforms into a 3D round cap, and the cap geometry can be precisely tuned by the radius ratio (r/R) in the original layout.

**Figure 6:**
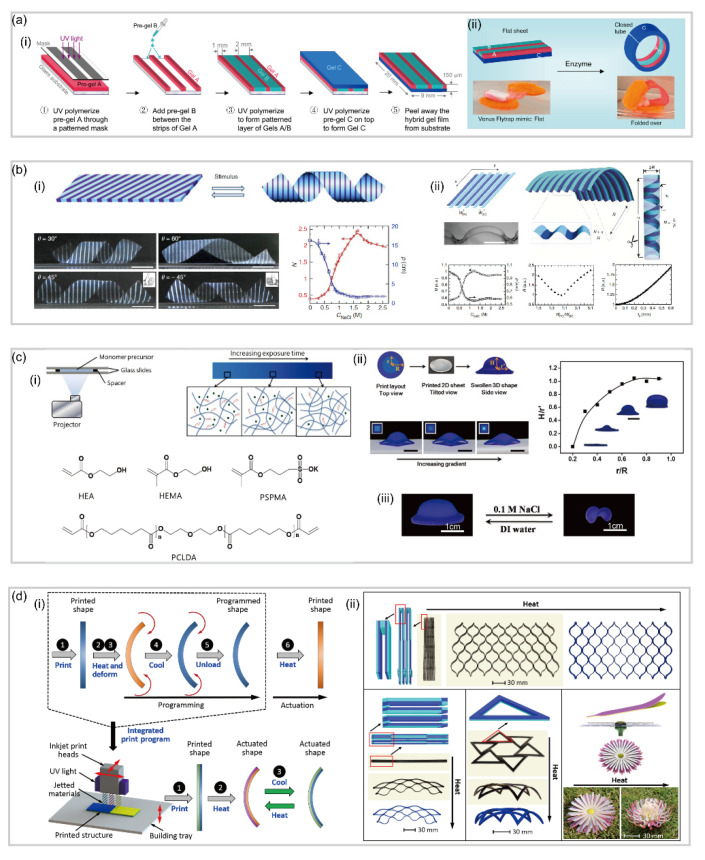
Pattern-based hydrogel soft robotics. (**a**) Fabrication of enzyme-responsive hydrogel actuator by UV-photolithography (reproduced with permission from Reference [72]). (**b**) Thermal responsive hydrogel actuator with periodic stripes (reproduced with permission from Reference [73]). (**c**) Experimental printing setup and process illustration from a planar sheet with patterned concentric circles swollen into a cap-shape 3D structure (reproduced with permission from Reference [76]). (**d**) The concept of direct 4D printing and its versatility in printing various configurations with a single material (reproduced with permission from Reference [77]).

Similarly, by employing 4D printing, Ding et al. [77] reported an approach to print composite polymers with 3D architectures which allows the object to transform into a new permanent shape by releasing the constraint on the strained elastomer of shaper memory polymer with time (the fourth dimension) after heating, which can then be reprogrammed into multiple subsequent shapes. They simplified the creation of high-resolution complex 3D reprogrammable structures by controlling the photopolymerization process during printing to enable 3D components with a complex geometric form at high spatial resolution. When removed from the build tray, those components exhibit high-fidelity features but with controlled built-in strains. The transformation step is also simply triggered by heating, and the shape remains stable in later variations in temperature (Figure 6d).

## 4. Outlook and Conclusions

In this review, we have presented the recent progress in the field of plant-inspired soft robotics fabricated by stimuli-responsive polymeric hydrogels. Applying the plant-inspired design principles of creating bilayer, gradient, and patterned anisotropic structures, hydrogel-based soft robotics achieving diverse deformations, such as bending, twisting, coiling, and folding, have been developed and presented (Table 1).

Though hydrogel-based soft robotics have demonstrated exciting and promising performances and application potentials, there are advantages and disadvantages for each kind of hydrogel prototype, to be more specific: (1) bilayer hydrogel-based soft robotics feature convenient preparation and low cost but less complicated actuation modes; (2) gradient hydrogel-based soft robotics tend to possess higher mechanical strength and diverse applications, like tissue repair, switch, and gripper, but limited methods for creating gradients in a single material; (3) patterned hydrogel-based soft robotics highly rely on the fabrication methods (i.e., layer-by-layer assembly and 4D printing) by which complex three-dimensional movements are realized.

To outlook, most applications of hydrogel-based soft robotics are still in the conceptual stage, and practical applications should be explored and demonstrated in the future. Secondly, hydrogel-based soft robotics exhibiting multifunctional properties by embedding functional fillers, such as carbon nanotubes and magnetic particles, are highly demanded. Thirdly, the mechanical properties of hydrogel-based soft robotics need to be properly evaluated and improved in terms of toughness and self-healing. Fourthly, the response time of hydrogel-based soft robotics needs to be shortened as the state-of-art response time still sits in the range of several seconds to hours. Finally, mathematic models guiding the robot design and precise control of actuation need to be developed, especially for the pattern-based hydrogel soft robotics. To summarize, hydrogel-based soft robotics represent an important part of soft robotics and could find meaningful applications in the near future.

## Figures and Tables

**Table 1:** Comparison of three types of hydrogel soft robotics.

Type	Reference	Hydrogel	Response Time	Tensile Strength (kPa)	Motion	Stimulus
Bilayer	[58]	pNIPAM, GO	16–24 s	83	Bending	Temperature
	[62]	pNIPAM, pDADMAC			Bending	Temperature, pH
	[63]	pNIPAM, (AAc-co-AAm)	~60 s		Bending	Temperature
	[64]	CS, CMC	240 s	62	Coiling	pH
Gradient	[66]	pNIPAM, GO	40 s		Bending	Photo
	[67]	NAGA, NAT, TRIS-acrylamide		410		Temperature
	[71]	pNIPAM, Laponite	20 s	290	Bending	Temperature
Pattern	[72]	PEGDA, GelMA-co-PEGDMA		20.3/1.8	Folding, twisting	pH, enzyme
	[73]	pNIPAM, AMPS	2 h		Twisting	Temperature
	[76]	HEA, HEMA, PSPMA, LA, HD			Bending, folding	Temperature
	[77]	TangoBLack+, VeroClear			Bending, folding	Temperature
